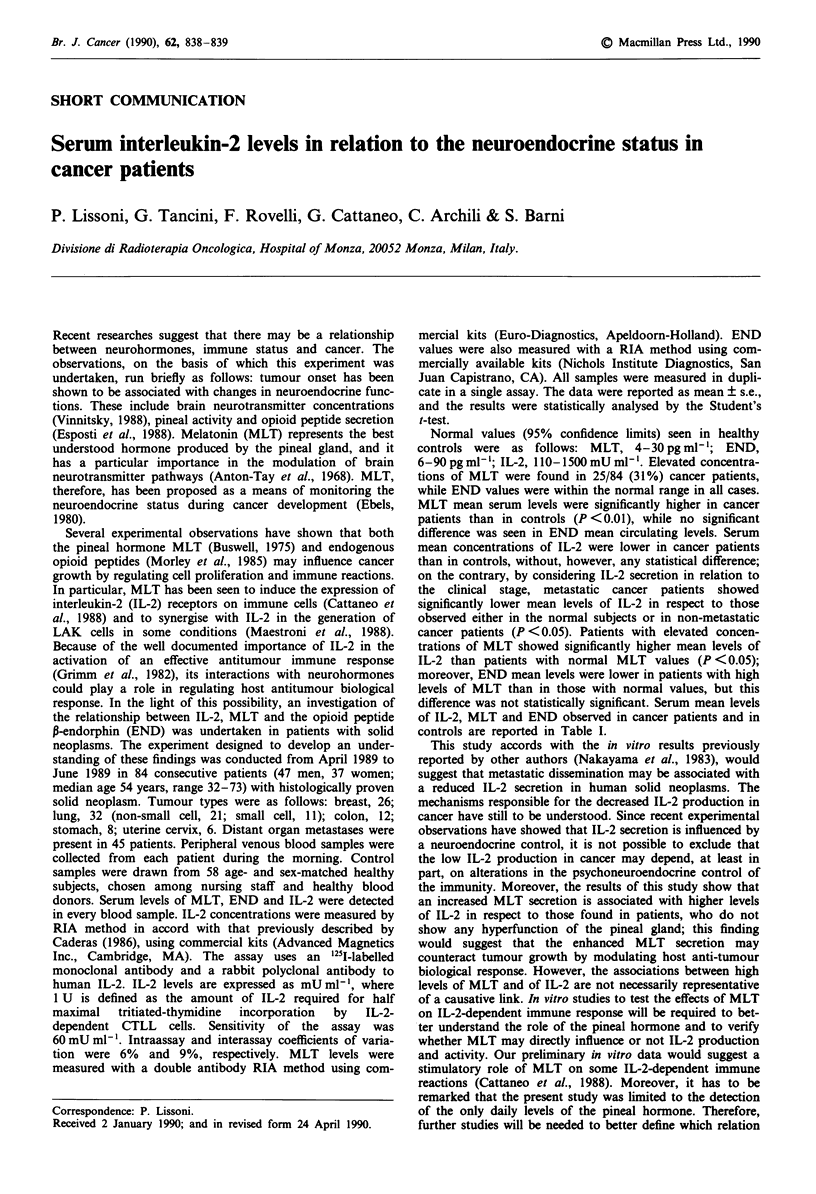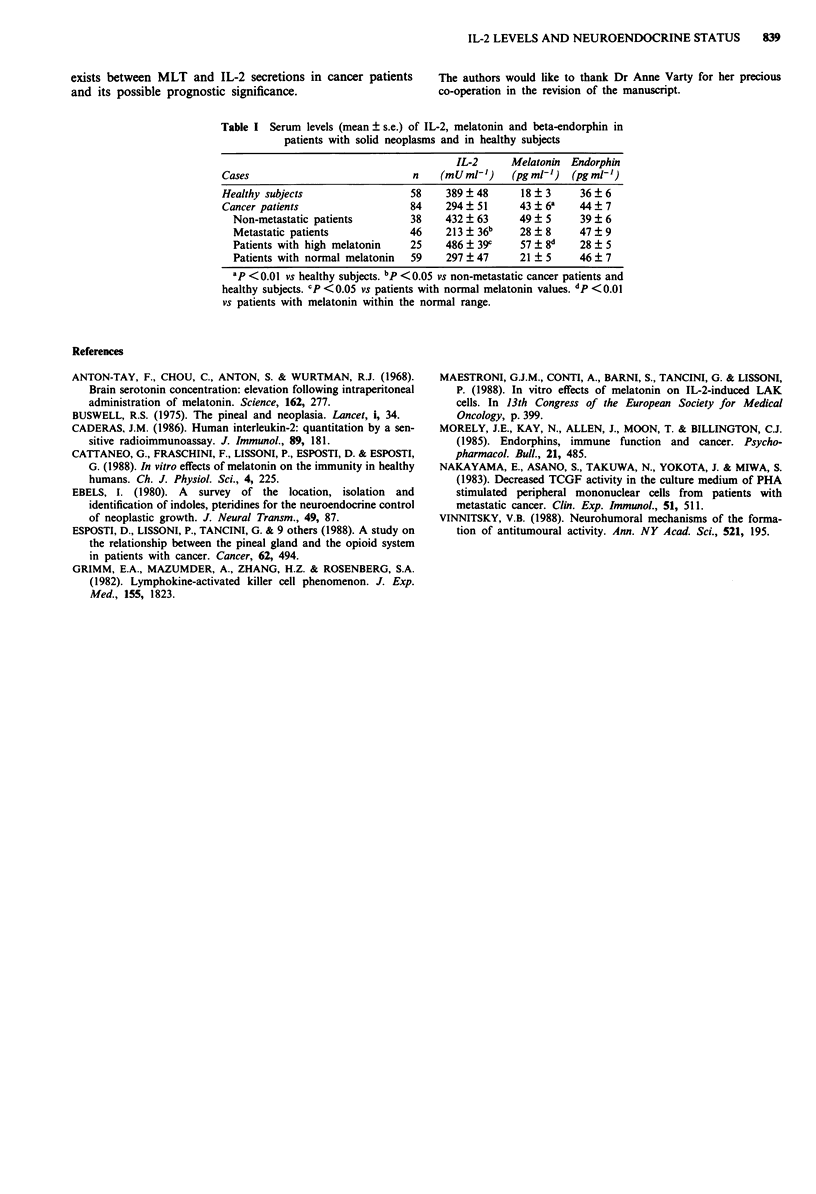# Serum interleukin-2 levels in relation to the neuroendocrine status in cancer patients.

**DOI:** 10.1038/bjc.1990.389

**Published:** 1990-11

**Authors:** P. Lissoni, G. Tancini, F. Rovelli, G. Cattaneo, C. Archili, S. Barni

**Affiliations:** Divisione di Radioterapia Oncologica, Hospital of Monza, Milan, Italy.


Br. J. Cancer (1990), 62, 838-839 © Macmillan Press Ltd., 1990

SHORT COMMUNICATION

### Serum interleukin-2 levels in relation to the neuroendocrine status in cancer patients

P. Lissoni, G. Tancini, F. Rovelli, G. Cattaneo, C. Archili & S. Barni

*Divisione di Radioterapia Oncologica, Hospital of Monza, 20052 Monza, Milan, Italy.*

Recent researches suggest that there may be a relationship between neurohormones, immune status and cancer. The observations, on the basis of which this experiment was undertaken, run briefly as follows: tumour onset has been shown to be associated with changes in neuroendocrine functions. These include brain neurotransmitter concentrations (Vinnitsky, 1988), pineal activity and opioid peptide secretion (Esposti *et al.*, 1988). Melatonin (MLT) represents the best understood hormone produced by the pineal gland, and it has a particular importance in the modulation of brain neurotransmitter pathways (Anton-Tay *et al.*, 1968). MLT, therefore, has been proposed as a means of monitoring the neuroendocrine status during cancer development (Ebels, 1980).

Several experimental observations have shown that both the pineal hormone MLT (Buswell, 1975) and endogenous opioid peptides (Morley *et al.*, 1985) may influence cancer growth by regulating cell proliferation and immune reactions. In particular, MLT has been seen to induce the expression of interleukin-2 (IL-2) receptors on immune cells (Cattaneo *et al.*, 1988) and to synergise with IL-2 in the generation of LAK cells in some conditions (Maestroni *et al.*, 1988). Because of the well documented importance of IL-2 in the activation of an effective antitumour immune response (Grimm *et al.*, 1982), its interactions with neurohormones could play a role in regulating host antitumour biological response. In the light of this possibility, an investigation of the relationship between IL-2, MLT and the opioid peptide β-endorphin (END) was undertaken in patients with solid neoplasms. The experiment designed to develop an understanding of these findings was conducted from April 1989 to June 1989 in 84 consecutive patients (47 men, 37 women; median age 54 years, range 32–73) with histologically proven solid neoplasm. Tumour types were as follows: breast, 26; lung, 32 (non-small cell, 21; small cell, 11); colon, 12; stomach, 8; uterine cervix, 6. Distant organ metastases were present in 45 patients. Peripheral venous blood samples were collected from each patient during the morning. Control samples were drawn from 58 age- and sex-matched healthy subjects, chosen among nursing staff and healthy blood donors. Serum levels of MLT, END and IL-2 were detected in every blood sample. IL-2 concentrations were measured by RIA method in accord with that previously described by Caderas (1986), using commercial kits (Advanced Magnetics Inc., Cambridge, MA). The assay uses an $^{125}$I-labelled monoclonal antibody and a rabbit polyclonal antibody to human IL-2. IL-2 levels are expressed as mU ml$^{-1}$, where 1 U is defined as the amount of IL-2 required for half maximal tritiated-thymidine incorporation by IL-2-dependent CTLL cells. Sensitivity of the assay was 60 mU ml$^{-1}$. Intraassay and interassay coefficients of variation were 6% and 9%, respectively. MLT levels were measured with a double antibody RIA method using commercial kits (Euro-Diagnostics, Apeldoorn-Holland). END values were also measured with a RIA method using commercially available kits (Nichols Institute Diagnostics, San Juan Capistrano, CA). All samples were measured in duplicate in a single assay. The data were reported as mean ± s.e., and the results were statistically analysed by the Student's *t*-test.

Normal values (95% confidence limits) seen in healthy controls were as follows: MLT, 4–30 pg ml$^{-1}$; END, 6–90 pg ml$^{-1}$; IL-2, 110–1500 mU ml$^{-1}$. Elevated concentrations of MLT were found in 25/84 (31%) cancer patients, while END values were within the normal range in all cases. MLT mean serum levels were significantly higher in cancer patients than in controls ($P < 0.01$), while no significant difference was seen in END mean circulating levels. Serum mean concentrations of IL-2 were lower in cancer patients than in controls, without, however, any statistical difference; on the contrary, by considering IL-2 secretion in relation to the clinical stage, metastatic cancer patients showed significantly lower mean levels of IL-2 in respect to those observed either in the normal subjects or in non-metastatic cancer patients ($P < 0.05$). Patients with elevated concentrations of MLT showed significantly higher mean levels of IL-2 than patients with normal MLT values ($P < 0.05$); moreover, END mean levels were lower in patients with high levels of MLT than in those with normal values, but this difference was not statistically significant. Serum mean levels of IL-2, MLT and END observed in cancer patients and in controls are reported in Table I.

This study accords with the *in vitro* results previously reported by other authors (Nakayama *et al.*, 1983), would suggest that metastatic dissemination may be associated with a reduced IL-2 secretion in human solid neoplasms. The mechanisms responsible for the decreased IL-2 production in cancer have still to be understood. Since recent experimental observations have showed that IL-2 secretion is influenced by a neuroendocrine control, it is not possible to exclude that the low IL-2 production in cancer may depend, at least in part, on alterations in the psychoneuroendocrine control of the immunity. Moreover, the results of this study show that an increased MLT secretion is associated with higher levels of IL-2 in respect to those found in patients, who do not show any hyperfunction of the pineal gland; this finding would suggest that the enhanced MLT secretion may counteract tumour growth by modulating host anti-tumour biological response. However, the associations between high levels of MLT and of IL-2 are not necessarily representative of a causative link. *In vitro* studies to test the effects of MLT on IL-2-dependent immune response will be required to better understand the role of the pineal hormone and to verify whether MLT may directly influence or not IL-2 production and activity. Our preliminary *in vitro* data would suggest a stimulatory role of MLT on some IL-2-dependent immune reactions (Cattaneo *et al.*, 1988). Moreover, it has to be remarked that the present study was limited to the detection of the only daily levels of the pineal hormone. Therefore, further studies will be needed to better define which relation

Correspondence: P. Lissoni.
Received 2 January 1990; and in revised form 24 April 1990.

exists between MLT and IL-2 secretions in cancer patients and its possible prognostic significance.

The authors would like to thank Dr Anne Varty for her precious co-operation in the revision of the manuscript.

**Table I** Serum levels (mean ± s.e.) of IL-2, melatonin and beta-endorphin in patients with solid neoplasms and in healthy subjects

| *Cases* | *n* | *IL-2 (mU ml$^{-1}$)* | *Melatonin (pg ml$^{-1}$)* | *Endorphin (pg ml$^{-1}$)* |
|---|---|---|---|---|
| *Healthy subjects* | 58 | 389 ± 48 | 18 ± 3 | 36 ± 6 |
| *Cancer patients* | 84 | 294 ± 51 | 43 ± 6[a] | 44 ± 7 |
| Non-metastatic patients | 38 | 432 ± 63 | 49 ± 5 | 39 ± 6 |
| Metastatic patients | 46 | 213 ± 36[b] | 28 ± 8 | 47 ± 9 |
| Patients with high melatonin | 25 | 486 ± 39[c] | 57 ± 8[d] | 28 ± 5 |
| Patients with normal melatonin | 59 | 297 ± 47 | 21 ± 5 | 46 ± 7 |

[a] $P < 0.01$ *vs* healthy subjects. [b] $P < 0.05$ *vs* non-metastatic cancer patients and healthy subjects. [c] $P < 0.05$ *vs* patients with normal melatonin values. [d] $P < 0.01$ *vs* patients with melatonin within the normal range.

### References

ANTON-TAY, F., CHOU, C., ANTON, S. & WURTMAN, R.J. (1968). Brain serotonin concentration: elevation following intraperitoneal administration of melatonin. *Science*, **162,** 277.

BUSWELL, R.S. (1975). The pineal and neoplasia. *Lancet*, **i,** 34.

CADERAS, J.M. (1986). Human interleukin-2: quantitation by a sensitive radioimmunoassay. *J. Immunol.*, **89,** 181.

CATTANEO, G., FRASCHINI, F., LISSONI, P., ESPOSTI, D. & ESPOSTI, G. (1988). *In vitro* effects of melatonin on the immunity in healthy humans. *Ch. J. Physiol. Sci.*, **4,** 225.

EBELS, I. (1980). A survey of the location, isolation and identification of indoles, pteridines for the neuroendocrine control of neoplastic growth. *J. Neural Transm.*, **49,** 87.

ESPOSTI, D., LISSONI, P., TANCINI, G. & 9 others (1988). A study on the relationship between the pineal gland and the opioid system in patients with cancer. *Cancer*, **62,** 494.

GRIMM, E.A., MAZUMDER, A., ZHANG, H.Z. & ROSENBERG, S.A. (1982). Lymphokine-activated killer cell phenomenon. *J. Exp. Med.*, **155,** 1823.

MAESTRONI, G.J.M., CONTI, A., BARNI, S., TANCINI, G. & LISSONI, P. (1988). In vitro effects of melatonin on IL-2-induced LAK cells. In *13th Congress of the European Society for Medical Oncology*, p. 399.

MORELY, J.E., KAY, N., ALLEN, J., MOON, T. & BILLINGTON, C.J. (1985). Endorphins, immune function and cancer. *Psychopharmacol. Bull.*, **21,** 485.

NAKAYAMA, E., ASANO, S., TAKUWA, N., YOKOTA, J. & MIWA, S. (1983). Decreased TCGF activity in the culture medium of PHA stimulated peripheral mononuclear cells from patients with metastatic cancer. *Clin. Exp. Immunol.*, **51,** 511.

VINNITSKY, V.B. (1988). Neurohumoral mechanisms of the formation of antitumoural activity. *Ann. NY Acad. Sci.*, **521,** 195.